# NGO perspectives on the challenges and opportunities for real-world evaluation: a qualitative study

**DOI:** 10.1080/16549716.2022.2088083

**Published:** 2022-09-14

**Authors:** Talata Sawadogo-Lewis, Ruth Bryant, Timothy Roberton

**Affiliations:** a Department of International Health, Johns Hopkins University Bloomberg School of Public Health, Baltimore, MD, USA; b Independent Consultant, Washington, DC, USA

**Keywords:** Evaluation, NGOs, donors, non-governmental organizations, maternal and child health

## Abstract

**Background:**

The move towards robust monitoring and evaluation (M&E) has been increasing in global health, motivated by both an accountability agenda and to increase learning from M&E activities. Many international non-governmental organizations (NGOs) receive funding from one or more large institutional donors.

**Objective:**

To understand NGOs’ perspective on their own role in terms of accountability to both donors and the populations they serve.

**Methods:**

We conducted a series of in-depth interviews with M&E staff in 11 NGOs with projects related to maternal and child health to better understand how M&E is being implemented in these organizations. We then examined the data based on *a priori* identified themes.

**Results:**

We found that despite flexibility from some donors, rigid reporting structures remain a barrier for NGOs to fully communicate the impact of their projects. While NGOs do utilize M&E findings, their use is limited by low staff capacity. The primary audience for the results remains the donor agency, and the primary motivation for M&E remains donor reporting. Reporting remains a burdensome affair, with ongoing limitations around streamlining results for donors. To reduce the burden of reporting for individual projects, the participants in our study suggested placing greater emphasis on process evaluations rather than impact evaluations. Participants also suggested increased data sharing between organizations working in the same regions and making better use of secondary data sources; in both cases to reduce the need for primary data collection.

**Conclusion:**

We carried out this work to advance the conversation on how NGOs currently manage their M&E – a conversation which should involve NGOs, donors, local health system actors, and the communities with whom they work. More flexibility from donors, increased use of technology, and more transparency on if and how data is being used would help NGOs with their M&E process.

## Background

Non-Governmental Organizations (NGOs) have become increasingly large players in the implementation of health sector intervention [1–3], and as such, are under increasing pressure to report to donors and the public on the implementation and impact of their programs [4]. NGOs have been celebrated as either flexible agents of change who directly address civil society’s needs without being encumbered by bureaucratic or corrupted governmental processes [5,6], or criticized as embodiments of cultural and economic colonialism [2]. Regardless of where NGOs lie on this spectrum, development through NGOs remains a significant and necessary contribution to the achievement of the sustainable development goal (SDG) of poverty eradication, along with other health-related SDGs [7]. Transparency and accountability of NGO work are crucial to increase the effectiveness of donor financial support, as well as to improve trust in NGOs [2,5,7].

Following heavy criticism about the perceived lack of NGO accountability in the 1990s, donors have since become increasingly focused on effectiveness, efficiency and transparency. The toolbox of financial audits, strategic planning, logic models, risk management and quantitative impact analysis became part of the donor-NGO relationship [6,8]. Logic models – a type of theory of change – have become increasingly part of donor requirements. At the program planning stage, they have the potential to clarify program objectives, mechanisms, and later can serve as an important tool for program evaluation [9]. Quantitative impact and process analysis has had the unintended consequence of a proliferation of indicators to meet specific donors’ requirements, which has in turn led to redundant reporting requirements and frustration from NGOs [10–12]. Indeed, resource allocation, project planning tools, teamwork and monitoring & evaluation were identified as key factors to improve the implementation of donor-funded projects in Kenya [13].

Given our own experiences in working within NGOs, we undertook a study to understand how NGOs currently carry out M&E activities, how this has evolved over time, and where they would like to see it go in the future. While other studies have looked at barriers and facilitators to the successful implementation of M&E within NGOs quantitatively [13–15], to our knowledge no study has looked at NGOs’ perceptions. This work is aimed at practitioners who work within NGOs, to advance the discussion within their organizations and with their donors. It is also aimed at donors attempting to address the challenges associated with the NGO-donor relationship.

## Methods

### Sampling

We used the donor’s online project browser which provides information about projects who receive funding from the donor. We carried out a search to find projects who (1) are currently operational, (2) have a partnership designation of ‘International NGO’ and/or ‘regional NGO’ and/or ‘national NGO’, and (3) come up for the search term ‘maternal’ and/or ‘child’ and/or ‘health’. This led to 46 results. We then reviewed the results and removed any projects that focused only on humanitarian aid and those who did not primarily focus on maternal and child health (5 projects).

Based on the distribution of funding amounts, we then categorized the remaining 41 projects into brackets of funding ([$0-1,000,000[, [$1,000,000–5,000,000[, [$5,000,000–10,000,000[, [$10,000,000–15,000,000, and [$15,000,000+]) and randomly selected 3 projects per bracket, to get a distribution of experiences by project size for a total of 12 sampled NGOs. We then contacted senior or mid-level staff in each organization’s research, monitoring, evaluation, learning, and/or accountability departments and carried out individual interviews with each over Zoom, given travel restrictions related to the novel coronavirus pandemic. Of note, organizations do not receive funding exclusively from this donor. Opinions expressed therefore may not reflect opinions related exclusively to this particular donor. Data collection took place between November and December 2020 and all NGOs included had active projects at that time.

### Data analysis

All interviews were recorded, and the interviewer took detailed notes. The researchers met weekly to discuss emerging themes and to adjust data collection on themes requiring further exploration. Once data collection was completed, we examined the data based on *a priori* identified themes, and report on those in this manuscript. The strength of this approach is that we were able to adjust data collection based on emerging themes. However, it also means that we may have missed some information based on the adjustments made along the way. All quotes included in this manuscript are taken verbatim from the participants themselves.

### Ethical considerations

All participants provided oral consent prior to the start of the interview. We also obtained ethical review and approval from the Johns Hopkins Bloomberg School of Public Health in (IRB00014275).

## Results

We interviewed a total of 13 individuals. We present our findings organized by our *a priori* themes with some supporting sub-themes below, namely: (1) donor and use of M&E data, (2) operating within a multi-level accountability system, (3) finding solutions to manage limited resources, (4) use of M&E findings and dissemination challenges, (5) trends in M&E over time, and (6) looking towards the future.

### Donor and use of M&E data

All participants unanimously pointed to their donor(s) as the primary audience for all M&E data, with participant 6 citing that *‘the donor is the number one client*’. This was generally expressed with some regret, as many participants expressed their belief that the audience should be wider. Depending on the structure of the organization, some then mentioned their own organization, or their local partners or country offices as secondary audiences. When stakeholders were included in the list of recipients of M&E data suggested by participants, they were listed last. Most participants immediately mentioned that reporting back to the communities should be a priority, but ultimately admitted that this was either not done, or done poorly.

Despite being the primary audience, participants noted that they did not get much feedback from the donor as to if and how the reported data is being used. Participant 6 spoke it thusly: ‘*it really sometimes sounds like you’re just pulling together information to feed into a machine, and I know to a certain extent that’s what it is*.’ Some participants brought this point even further, mentioning that not only is it not clear how this data is used in an immediate way, but it is also unclear how this data is used to inform policy decisions or decisions around funding allocation more generally.

In terms of collaboration on M&E, the donor imposes the format of the reporting (one performance management framework which assesses quantitative progress towards outputs and outcomes, and a narrative report). Many also mentioned that the donor initially suggested a large number of indicators, with participant 5 putting it as: ‘*the number of indicators that we needed to report on was astronomical, and so to me I think they were really great nice-to-haves for them, for their priorities but they didn’t take into account the pressure that would be on the teams to collect that data, or the fatigue in communities*’. However, all participants recognized that the donor provided ample room for discussion in terms of the actual selection of the indicators to measure. Participant 8 suggested that it was the responsibility of the organization to advocate for fewer indicators, phrased it as: *‘at the end of the day [the donor] wants to know specific things, but it’s also up to the implementing organization to share whether relevant for the project, and if it’s feasible*’.

Participants were unanimous in advocating for more qualitative data to be collected, and for it to be more valued by the donor. The reporting structure with this donor does already make a provision for a narrative to accompany quantitative data. However, participants listed many benefits that they know their projects have (e.g. individual stories of financial empowerment, increased collaboration with communities, increased knowledge about specific topics), but which cannot be quantified. Because these stories are often unique and specific, participants recognized that trying to report against a structured reporting frame is challenging, with participant 10 mentioning: ‘*I’m still looking for ways to measure qualitative indicators in a way that will please the reporting mechanism*.’

### Operating within a multi-level accountability system

Participants generally recognized that accountability to the donor is justified, with participant 10 illustrating as: ‘*we’re responsible for the money we receive, and we should make the difference [sic] with the money*’. NGOs have a clear incentive to show that their projects and programs are leading to impact, given the competitive nature of the funding landscape. However, some participants mentioned that the donor is open to receiving honest accounts of the project, with participant 2 phrasing it as: ‘*we try to be forthright and transparent and certainly when there are problems and failures, [the donor] is open to that […] we don’t try to make things look better than they are*.’

Additionally, participants recognize that their donors are themselves accountable to their own stakeholders, and therefore understand the need for reporting in a way that allows donors to report back to their population in turn. This second level of accountability focuses more on the actual impact of the projects than any project implementation metrics, with participant 9 phrasing it as: ‘*it’s a fairly simple answer, and it’s for political purposes, so the politicians can say “our program directly saved the lives of 5,000 children or whatever”*’. This limits the type and number of M&E activities and is seen as the primary reason why M&E continues to be more quantitative, with participant 7 expressing it as: ‘*Learned lessons are presented as opportunities, but no one has time to use these opportunities […] it’s sort of lip service*’.

In general, while many participants had comments about their projects being underfunded or feeling constrained by their budgets in general, this did not extend to M&E funding specifically. Participant 11 exemplifies it as: ‘*it’s not a challenge with the donor, generally we get an envelope of funds and we need to budget it, and we provide a budget that includes the [Monitoring Evaluation Research and Learning] activities*’. However, other participants spoke to the difficulty in ensuring that these dedicated funds do get used for M&E within the organization. Having a donor which places high importance on M&E helped with being able to defend M&E interests in-house.

In terms of reporting mechanisms, most participants mentioned that having clear expectations from their donor is helpful for them to orient the direction in which they take M&E. Given that most interactions with the donor go through the project officer assigned to that project, high turnover in the role has made continuity difficult. Additionally, conflicting guidelines from different factions of the donor agency are hard to consolidate, with participant 6 illustrating it in this way: ‘*one person at [the donor agency] said this “wow, this is really great, it’s really interesting”, while another person at [the donor agency] said “yeah … that’s a lot of effort for just 3 indicators”. So, it really varies’*. Relatedly, a recurring criticism was the disconnect between project officers and the reality of the field, with some participants highlighting that the difference between officers who have worked in the field and those who have not is clear in terms of how bureaucratic the process of interacting with them is.

While reporting is described as tedious, it also forces a moment of reflection for the organization which might not otherwise occur. Additionally, the inclusion of specific indicators automatically means that effort must go towards achieving progress towards this indicator. Participant 10 highlights this as a positive aspect in the context of gender indicators:

‘*the [donor] has forced us to invest in terms of results that modify conditions for women. It’s powerful for that. It forces to change your mindset; it forces you to get a good grade in terms of gender transformation*’

### Finding solutions to manage limited resources

Despite recognizing that securing funds for M&E from the donor was generally achievable, ensuring that the funds within the organization are indeed used for M&E remains a challenge. This was reported as generalized funding limitation, with organizations feeling constrained. To address this, some participants noted that securing a budget sufficient to carry out their M&E activities was more successful when they were able to involve senior leadership in M&E activities, and give them a sense of the importance of M&E. Participant 11 exemplifies it as: ‘*this comes back to how we need to involve leadership and management on the requirements of the MERL system and what that means in terms of the MERL budget. Based on recent examples, it continues to be a challenge for people to understand how much budget is required*.’

Participants mentioned that having insufficient staff capacity is an ongoing challenge to carrying out quality M&E. In smaller organizations, M&E activities tend to not be assigned to dedicated staff or to a department but rather be tacked on as an extra responsibility for project managers. Staff turnover also presents a challenge to ensuring continuity and uniformity in data collection and in reporting. Participant 10 spoke to this scenario: ‘*you collect the data at baseline and then the year after people forgot how they collected the data, and they change the definition of the indicator, the numerator, the denominator […] the team is a bit too mobile, so if you don’t write this at the beginning […] you find that a year later it’s not collected in the same way*’. To increase staff capacity, participants spoke to utilizing training opportunities as often as possible, be it formal training provided by the donor, or coaching and mentorship provided within the organization.

The structure of M&E varies by organization, with larger organizations tending towards having designated M&E staff or even M&E departments. However, regardless of the formal structures, initiatives that integrate M&E within the organization – rather than treating it as a silo – seem to be most successful to ensuring that M&E is valued and utilized. In particular, having a champion at the senior level who advocates for stronger M&E within the organization makes it easier to ensure that M&E become or remains prioritized.

Overburdened staff has led many organizations to rely on external consultants to support M&E activities. This has had mixed results, with concerns ranging from low capacity in the consultants hired, to fact that the skills and expertise is not kept in-house and is therefore lost once the consultant is changed. Even when collaborating with consultants, the final responsibility still lays with the M&E staff at the NGO, which can still be a significant weight on internal staff. Another result of the fast-paced deadlines and turnaround times is that it limits the amount and ways that participants can engage with local partners in M&E.

### Use of M&E findings and dissemination challenges

All participants expressed that M&E data is generally seen as valuable within their organization, primarily to help inform programmatic decisions. Indeed, some participants expressed that because reporting is a mandatory requirement, it forces the organization to pause and introspect, as participant 6 mentions: ‘*for us to see: are we doing the right thing? Are we doing it the right way? Are we meeting the right people? Are we meeting their needs*?’. A secondary use of M&E data is to help with communications and fundraising for the organization more generally. Armed with accurate data, organizations can share actual accomplishments, as participant 8 puts it: ‘*it helps in sharing results that are really reliable, that are real. It’s not just “we reached students”, it’s how many of them, where, on which topic, and that really helps in raising awareness […] and helps with fundraising*.’

One challenge to using M&E findings is that it is not always shared in a format that is easy to use for different audiences. This can mean that it is literally not translated to the right language for local partners and communities to be able to read it, or that it uses technical terminology that make it difficult for various partners and populations to understand it. For those working with more complicated quantitative data analysis, quantitative data must be shared in a way that is comprehensible to people beyond simply the data manager.

To increase uptake and understanding of the data, dissemination events serve an important role. Carrying out data analysis or data interpretation workshops either internally or with external stakeholders is described as a useful mechanism to both help contextualize findings and conversely to share findings with stakeholders in a way that is more engaging than a report. However, these dissemination events tend to be the first ones to be skipped when crunched for time or budget.

In terms of the concrete mechanisms for data management, most organizations use Excel spreadsheets as their primary way of storing and sharing data within the organization. Others mentioned that some data comes from local partners in a strictly narrative format in a Word document, and some also just via email. Embracing technology for data collection using applications such as Open Data Kit (ODK – https://opendatakit.org/) and KoBoToolbox (https://www.kobotoolbox.org/) has simplified data collection for some organization, but the workload for data analysis and management continues to be a challenge. Overall, consolidating all the data in a format that is cohesive and to report to the donor is a heavy challenge.

For larger NGOs who need to collate data from multiple country offices, collating data in a uniform way can be challenging. In general, this process is not quite systematized, and requires a good amount of back and forth with country offices or local partners. Some participants pointed to the rigid structure of quantitative reporting not allowing to capture the full picture, while others simply mentioned that their organization has no other system in place to facilitate this process. To illustrate the first point, participant 2 mentioned:

‘*we’ll get one report [from the country coordinator] saying there’s 50 women doing [an activity], and 6 months later we get another report and it’ll say 120 women doing; we’ll say ‘oh that’s great, it looks like an increase’ and the partner will say ‘not really, because of those 50, 20 had to abandon their [activity], some are from the old villages, some of them are new participants and some are the same as last year […] do you want to tell you the new ones and old ones together?’*

### Trends in M&E over time

Over the past decade, participants report noticing a shift in how M&E is perceived – moving from a more punitive or regulatory role to more of a collaborator, as explained by participant 2:

‘*So it became friendly and popular, and it’s not seen as ‘oh you’re the evaluator, you’re here to tell us what’s wrong’ but mostly like ‘ok, we’re doing this together, M&E is going to help us internally’ – people really see the value*’

Participants credit the push towards results-based management (RBM) – a donor requirement consisting in reporting against targets set on the basis of achieving results – for both increasing the rigor of M&E, and for increasing the burden associated with achieving this rigor. Generally, the focus on results is seen positively in theory, with participants understanding the advantages of going beyond intermediary outcomes. However, this new structure has also led to heavier reporting demands from the donor, with participant 3 mentioning: ‘*[…] there is certainly more ambition for M&E. I do see a move for more rigor. I don’t think that’s always aligned with what’s required or what’s feasible, but the ambition is there, more and more […] but sometimes those ambitions … they don’t work*’. Additionally, most participants emphasized that understanding both context and process needed to be prioritized, and that the focus on the results does not allow this to be captured. For example, Participant 4 suggests the following:

‘*I think that maybe focus more on the quality of implementation, on how well you’re delivering the service […] and maybe leave the more standardized indicator for post-implementation evaluations. That might fit well within a region or at national level or using secondary data … you can be creative in terms of how to think about that, but not burden the individual project in terms of having that level of outcome*’

### Looking towards the future

Participants’ calls for more flexibility in reporting were frequently repeated. Participants requested making provisions for the realities of the field and allowing for course correction along the way. This was mentioned both for data collection exercises as well as for adjusting the theory of change over the course of the project. As participant 3 mentions: ‘*rarely does a project plan go seamlessly and exactly to plan – making adjustments along the way is part of being a practitioner*’.

Participants expressed a need for increased data sharing across different organizations, which could potentially reduce the M&E burden of each individual project or program, with participant 9 suggesting: ‘*in a better world, there would be increased sharing of data between organizations*. […] *with an increased movement towards open data and data sharing, it would be good if we move in that direction*’. Another approach is to move towards using secondary data sources, although participants who have tried or are currently using this approach describe challenges around data quality and data use permissions.

The novel coronavirus (COVID-19) pandemic and the associated limitations on travel has meant that all organizations are now relying more heavily on alternative solutions to achieve results remotely. This has created an opportunity for examining how M&E can be done. As participant 3 reports: ‘*Well the COVID-19 pandemic has certainly put interesting new pressures on M&E to innovate. I think there will be more and more more tech solutions that people will be looking to facilitate M&E […] GIS mapping, mobile data collection apps, other tech-based phone apps, interactive apps to interact with participants at a safe distance*’. Relatedly, participants also foresaw an upcoming reduction in funding due to the economic impact of the COVID-19 pandemic and suspect that funds for M&E will be the first to be reduced in the face of urgent need from populations.

## Discussion

Our findings are likely not surprising to anyone working within the donor-funded NGO setting. While there are promising findings – such as this funder’s willingness to fund M&E, and flexibility for indicator selection – the general sentiment remains that M&E is heavy, burdensome work that is still underutilized. An ongoing challenge for evaluation more generally is defining and quantifying success for an organization, which should include clear objectives and involvement of stakeholders throughout the M&E process [14,16]. Indeed, participants unequivocally spoke to the need to include more qualitative data, whether systematically collected or simply through reporting anecdotes.

As participants mention, the activities that would allow organizations (i.e. data analysis or interpretation workshops, reflection sessions, dissemination events, etc.) to best utilize the data that they collect are often the first ones to be skipped when resources are too limited. Importantly, the findings from these conversations confirm that donors have the capacity to orient the work that they fund, which has also been found elsewhere [17]. If donors choose to prioritize learning in NGOs (by adding indicators pertaining to this in measurement plans, for example), then the organizations will be forced to create the space and opportunities for this learning to happen. Adjusting donor requirements based on project length and level of funding might also be a way for donors to alleviate reporting requirements for NGOs. Finally, providing insight as to whether and how the data that NGOs painstakingly collect is used beyond fulfilling reporting requirements might help NGOs connect to the greater effort.

While the trend towards increasing use of technology holds potential, assuming that this will automatically lead to a reduced burden on M&E staff is less clear. Indeed, implementing new systems is challenging, and while technology can help simplify certain aspects of data management, it cannot singlehandedly address all issues. Indeed, the learning curve associated with new technology has been identified as a barrier to the use of M&E digital systems elsewhere [15]. The human element in all structures involved must be considered, and ensuring that projects are sufficiently staffed and provided with training as necessary will continue to be a priority. This finding echoes those of Micah & Luketero [15], who also found that appropriately trained staff equipped with sufficient time resulted in more efficient M&E within NGOs [15]. Additionally, given the calls for collecting more qualitative data, careful consideration must be given to how, when, and in what format digital solutions are most appropriate.

Moving towards an open-source data-sharing platform where data could be shared across any organization working in the same region holds potential. Donors would be best placed to serve as data custodians, able to host and point grant-recipients to relevant existing data. However, questions around data ownership (particularly of data collected in other countries), data quality, comparability of data are challenges to consider. In the meantime, donors could play a role in facilitating conversations among M&E practitioners and ensuring that a community of practice continues to thrive.

### Limitation

One limitation to this study is the use of a single donor database to identify NGOs. We chose this methodology to have a framework from which to identify different types of NGOs and project sizes. We also sampled individuals who were responsible for M&E in their organization, and who therefore are very familiar with the M&E processes within their organization. However, their position may also have influenced how they perceive the value and importance of M&E overall. Additionally, we chose to focus on maternal and child health for this particular study, which might also limit our generalizability of the findings. Also, the participants were based out of the organization that received direct funding from the donor. In this case, all participants were based out of the head offices, which again limits the generalizability of findings

## Conclusion

In conclusion, finding the delicate balance between rigorous and sound M&E and what is feasible and actionable remains a challenge. More flexibility from donors, increased use of technology, and more transparency on if and how data is being used would help NGOs with their M&E process. Additionally, more information sharing – including of failures and lessons learned – across NGOs could help reduce duplication and allow better use of existing M&E data. Participant 6 summarizes the overarching challenge best: ‘We have to find that nexus where it’s interesting, where it’s useful, where it makes sense to all the right people involved to be able to move forward’.

## Paper context

Increasing attention is being paid to monitoring and evaluation in the global health arena – particularly within international non-governmental organization. However, little research has been done on these organizations’ own perception of their role and challenges. This paper shines light on what is working well, what is working poorly and what could be improved from their perspective. These insights serve to advance the discussion on how to improve accountability to both donors and populations.
